# Investigating the role of dystrophin isoform deficiency in motor function in Duchenne muscular dystrophy

**DOI:** 10.1002/jcsm.12914

**Published:** 2022-01-26

**Authors:** Mary Chesshyre, Deborah Ridout, Yasumasa Hashimoto, Yoko Ookubo, Silvia Torelli, Kate Maresh, Valeria Ricotti, Lianne Abbott, Vandana Ayyar Gupta, Marion Main, Giulia Ferrari, Anna Kowala, Yung‐Yao Lin, Francesco Saverio Tedesco, Mariacristina Scoto, Giovanni Baranello, Adnan Manzur, Yoshitsugu Aoki, Francesco Muntoni

**Affiliations:** ^1^ Dubowitz Neuromuscular Centre UCL Great Ormond Street Institute of Child Health London UK; ^2^ Population, Policy and Practice Research and Teaching Department UCL Great Ormond Street Institute of Child Health London UK; ^3^ NIHR Great Ormond Street Hospital Biomedical Research Centre UCL Great Ormond Street Institute of Child Health London UK; ^4^ Department of Molecular Therapy National Institute of Neuroscience, National Center of Neurology and Psychiatry (NCNP) Kodaira Japan; ^5^ Department of Cell and Developmental Biology University College London London UK; ^6^ Centre for Genomics and Child Health, Blizard Institute, Barts and the London School of Medicine and Dentistry Queen Mary University of London London UK; ^7^ The Francis Crick Institute London UK

**Keywords:** Duchenne muscular dystrophy, Isoform, Motor function

## Abstract

**Background:**

Duchenne muscular dystrophy (DMD) is caused by *DMD* mutations leading to dystrophin loss. Full‐length Dp427 is the primary dystrophin isoform expressed in muscle and is also expressed in the central nervous system (CNS). Two shorter isoforms, Dp140 and Dp71, are highly expressed in the CNS. While a role for Dp140 and Dp71 on DMD CNS comorbidities is well known, relationships between mutations expected to disrupt Dp140 and Dp71 and motor outcomes are not.

**Methods:**

Functional outcome data from 387 DMD boys aged 4–15 years were subdivided by *DMD* mutation expected effects on dystrophin isoform expression; Group 1 (Dp427 absent, Dp140/Dp71 present, *n* = 201); Group 2 (Dp427/Dp140 absent, Dp71 present, *n* = 152); and Group 3 (Dp427/Dp140/Dp71 absent, *n* = 34). Relationships between isoform group and North Star ambulatory assessment (NSAA) scores, 10 m walk/run velocities and rise time velocities were explored using regression analysis.

Western blot analysis was used to study Dp427, Dp140 and Dp71 production in myogenic cells (control and DMD human), control skeletal muscle, DMD skeletal muscle from the three isoform groups and cerebral cortex from mice (wild‐type and DMD models). Grip strength and rotarod running test were studied in wild‐type mice and DMD mouse models. DMD mouse models were *mdx* (Dp427 absent, Dp140/Dp71 present), *mdx52* (Dp427/Dp140 absent, Dp71 present) and *DMD‐null* (lacking all isoforms).

**Results:**

In DMD boys, mean NSAA scores at 5 years of age were 6.1 points lower in Group 3 than Group 1 (*P* < 0.01) and 4.9 points lower in Group 3 than Group 2 (*P* = 0.05). Mean peak NSAA scores were 4.0 points lower in Group 3 than Group 1 (*P* < 0.01) and 1.6 points lower in Group 2 than Group 1 (*P* = 0.04).

Mean four‐limb grip strength was 1.5 g/g lower in *mdx52* than *mdx* mice (*P* = 0.003) and 1.5 g/g lower in *DMD‐null* than *mdx* mice (*P* = 0.002).

Dp71 was produced in myogenic cells (control and DMD human) and skeletal muscle from humans in Groups 1 and 2 and *mdx* mice, but not skeletal muscle from human controls, myogenic cells and skeletal muscle from humans in Group 3 or skeletal muscle from wild‐type, *mdx52* or *DMD‐null* mice.

**Conclusions:**

Our results highlight the importance of considering expected effects of *DMD* mutations on dystrophin isoform production when considering patterns of DMD motor impairment and the implications for clinical practice and clinical trials. Our results suggest a complex relationship between dystrophin isoforms expressed in the brain and DMD motor function.

## Introduction

Duchenne muscular dystrophy (DMD) is an X‐linked recessive condition caused by *DMD* mutations leading to dystrophin deficiency.^1^, ^2^ DMD boys typically present with delayed motor milestones, frequent falls and speech delay.^2^ Neurobehavioural comorbidities, including intellectual disability, attention deficit hyperactivity disorder and/or autism spectrum disorder, occur in approximately one third of patients.^2^ Loss of ambulation (LOA) typically occurs by 12 years of age, followed by cardiomyopathy and respiratory insufficiency.^2^ Life expectancy is reduced to around the thirties.^3^

Duchenne muscular dystrophy shows significant clinical heterogeneity. Some heterogeneity is related to *DMD* genotypes allowing low level dystrophin production.^4^ Gene modifiers other than *DMD* have recently been identified.^5^, ^6^, ^7^ However, each modifier only modestly contributes to DMD heterogeneity, suggesting other, as yet unidentified, factors.

The *DMD* locus encodes multiple isoforms.^8^ Full‐length Dp427 (427 represents molecular weight in kDa) exists in three isoforms. Dp427m is expressed highly in skeletal muscle and, together with Dp427c and Dp427p, at low levels in human brain.^8^, ^9^ Shorter dystrophin isoforms are driven by intronic promoters further downstream the gene. Dp140 and Dp71 are abundantly expressed in brain, with Dp71 having a broader pattern of expression, including liver, testis, lung and kidney.^8^, ^10^, ^11^ While in DMD all mutations affect Dp427 production, the *DMD* mutation location can also disrupt production of one or both of Dp140 and Dp71. Dp260 and Dp116 expression is limited to retina and peripheral nerves respectively.^8^

Several studies have suggested a role of Dp140 and Dp71 in DMD central nervous system (CNS) involvement.^12^, ^13^, ^14^, ^15^, ^16^, ^17^, ^18^ 15% of DMD boys lacking only Dp427 had intellectual disability, compared with 25% of boys lacking Dp427 and Dp140 and 64% of boys lacking Dp427, Dp140 and Dp71.^18^

We hypothesized that *DMD* mutations expected to disrupt different dystrophin isoforms may have a different impact on DMD motor outcomes. We conducted a series of studies addressing this. We evaluated relationships between *DMD* mutations expected to lead to loss of Dp427/Dp140 and Dp427/Dp140/Dp71 compared with loss of Dp427 alone on motor function in a large cohort of DMD boys. We focused on the following motor outcomes commonly used in clinical trials: (1) North Star ambulatory assessment (NSAA) score, (2) rise from supine time and rise from supine time velocity, (3) 10 m walk/run (10MWR) time and velocity and (4) age at LOA. We conducted parallel experiments in three DMD mouse models with mutations leading to differential involvement of the three isoforms. Finally, we evaluated dystrophin isoform protein production in human control and DMD (from the three isoform groups) skeletal muscle and myogenic cells in culture, and in muscle and brain of wild‐type and three DMD mouse models.

## Methods

## Duchenne muscular dystrophy boys

### Study design and participants

Three hundred eighty‐seven participants aged 4–15 years with DMD or intermediate muscular dystrophy (IMD) and an out‐of‐frame *DMD* deletion or duplication were included. Participants with in‐frame *DMD* deletions/duplications and IMD, Becker muscular dystrophy, female carriers and clinical trial participants were excluded. Participants were recruited from the North Star network of UK centres looking after DMD patients.^19^, ^20^ Clinical data, collected six monthly using standard operating procedures, is stored, with written consent, in an electronic database managed by CertusLtd. The NSAA is a 17‐item DMD‐specific motor function scale with maximum total score of 34.^21^

### *DMD* mutation data

Participants were grouped into 3 groups based on predicted *DMD* mutation effects on dystrophin isoform expression: Group 1 (Dp427 absent, Dp140/Dp71 present, *n* = 201); Group 2 (Dp427/Dp140 absent, Dp71 present, *n* = 152); and Group 3 (Dp427/Dp140/Dp71 absent, *n* = 34). Participants with *DMD* mutations only involving the region upstream of intron 44 were considered Dp427 negative and Dp140/Dp71 positive (Group 1), participants with *DMD* mutations involving the region from exon 51 to exon 62 inclusive and not involving the region of exon 63 or downstream of exon 63 were considered Dp427/Dp140 negative and Dp71 positive (Group 2) and participants with *DMD* mutations involving exon 63 and/or the region downstream of exon 63 were considered Dp427/Dp140/Dp71 negative (Group 3).^10^, ^22^ Participants with *DMD* mutations involving exon 45 to exon 50 inclusive and not involving the region of exon 51 and downstream of exon 51 were excluded from analysis due to difficulties predicting their effects on Dp140 expression as the Dp140 promoter is in intron 44 and its translation start site in exon 51.^10^, ^22^

### Cognition grouping

Cognitive status was evaluated by formal testing of intelligence quotient (IQ) in a subset of 40 boys who have participated in other previously reported studies at our centre^16^, ^23^ or presence/absence of learning difficulties as determined by parental and/or physician and/or educational report. The cognitive impairment group had learning disabilities [as reported by parent/guardian(s) and/or physician and/or educational services] and/or an IQ of less than 85 (>1 standard deviation below mean population IQ).^24^, ^25^ The normal cognition group had no learning disability [as reported by parent/guardian(s) and/or physician and/or educational services] and/or an IQ of 85 or above.

### Statistical analysis

Patient characteristics were summarized using mean and standard deviation for continuous data and frequency and proportion for categorical data. Glucocorticoid (GC) regimen was summarized as the regime (daily, intermittent/other or none) taken for the longest duration over both the full longitudinal period (majority regimen) and the duration prior to peak NSAA (early regime). Where age of GC initiation was not available, this was estimated based on mean age of GC initiation in the cohort. Ambulatory function at 5 years was described for patients with a visit between 4.5 and 5.5 years of age.

Comparisons between isoform and cognitive impairment groups were made using one‐way analysis of variance and *χ* ^2^ tests. For NSAA score, 10MWR velocity and rise time velocity, relationships with age were explored for each isoform group; additionally, relationships for NSAA score with age were explored in the two cognition groups. Fractional polynomial regression, accounting for the longitudinal data, was used to find best fitting models by comparing model deviances.^26^ Noting the different mean peak functional scores for the isoform and cognition groups, subsequent analysis focussed on the age range 5.5–8 years, when we expected most participants to reach peak motor function, calculating age and magnitude of peak observed motor function.

Multivariable regression analysis was used to compare maximum scores between isoform and cognition groups. We assessed whether differences in GC early regime accounted for differences between isoform groups. Kaplan–Meier survival estimation was used to estimate median time to LOA for each isoform group. *P* value ≤0.05 was considered statistically significant.

Statistical analysis and figure creation for DMD patient analysis was conducted by Dr Deborah Ridout in Stata v 15 StataCorp. 2017. *Stata Statistical Software*: *Release 15*. College Station, TX, USA: StataCorp LLC.

## Human pathology studies

We studied Dp71 protein expression in normal and DMD skeletal muscle biopsies using Western blot and immunohistochemistry. See *Table* S1 for sample details. We also studied Dp71 expression in myogenic cells derived either by myogenic conversion of dermal fibroblasts from skin biopsies following transduction by a lentiviral‐mediated MyoD construct carrying a puromycin selection cassette (for transduced cell enrichment) and a DsRed cassette (for assessing transduction efficiency) or from human‐induced pluripotent stem cell (iPSC)‐derived myogenic cells. Human iPSC‐derived myogenic cells are outlined in *Table* S1 [Sample 13^27^ and sample 14 (hpscreg.eu line UCLi011‐A).^28^ Sample 14 was differentiated as previously described^29^].

Proteins from control and DMD cells and muscles were solubilized in lysis buffer (urea 4 M, Tris 125 mM pH 6.8, SDS 4%) containing protease and phosphatase inhibitors (Roche, Merck, Hertfordshire, UK). Protein concentrations were measured using the Pierce BCA Protein assay kit (Thermo Scientific 23225), according to manufacturer instructions.

Capillary Western immunoassay (Wes) analysis was performed on a Wes system (ProteinSimple) according to manufacturer instructions using a 66–440 kDa Separation Module (ProteinSimple). For dystrophin, a rabbit polyclonal anti‐dystrophin antibody (ab15277, dilution 1/50 or ab154168, epitope dilution 1:1000, both from Abcam) and an anti‐rabbit secondary antibody (042‐206, Protein Simple) were used.

For immunocytochemistry, frozen muscle sections were stained with an antibody against the C‐terminus (Dys2, Leica Biosystem, UK) according to standard procedures followed in the diagnostic laboratory.^30^

Patients and controls provided written informed consent for biopsy samples. Fibroblasts, iPSCs and muscle tissues were supplied by the MRC Centre for Neuromuscular Disease Biobank London (REC reference number 06/Q0406/33). The human pathology studies were conducted under the REC reference numbers 13/LO/1894 and 13/LO/1826.

## Mouse studies

Mice used in this study were maintained at the National Center of Neurology and Psychiatry (NCNP).^31^ Three DMD mouse models were studied; *mdx* (Dp427 absent, Dp140/Dp71 present), *mdx52* (Dp427/Dp140 absent, Dp71 present) and *DMD‐null* (lacking all dystrophin isoforms).^31^, ^32^, ^33^ *Mdx52* mice were kindly provided by Dr T. Sasaoka (Brain Research Institute, Niigata University, Niigata, Japan).^33^ *DMD‐null* mice were generated by Dr Kazunori Hanaoka.^32^ C57BL/6 control mice were used to match the C57BL/6 background of the 3 DMD mouse models. Genotyping was performed using the previously described PCR method.^31^, ^32^, ^33^ Animal care was provided by the NCNP Small Animal Research Facility. Mice were allowed ad libitum access to food and drinking water. All behavioural experiments were performed between 9:00 am and 1:00 pm in strict accordance with the regulations of the National Institute of Neuroscience and the National Center of Neurology and Psychiatry (Japan) for animal experiments and were approved by the institute's Animal Investigation Committee. GraphPad Prism8 (GraphPad Software Inc., La Jolla, CA, USA) was used for statistical analysis and figure creation.

### Four‐limb grip strength

The grip strength test was conducted on wild‐type, *mdx*, *mdx52* and *DMD‐null* (*n* = 10 for each group) mice at 3 months of age. Mice were tested to determine peak paw grip strength, positioned horizontally from a grip bar using a grip strength meter (MK‐380 M; Muromachi Kikai Co., Ltd., Japan) as previously described, and pulled back slowly and steadily until mice released their grip.^34^ This was repeated six times, and peak force for the four‐limb paws was measured. Grip strength was normalized for mouse body weight measured on the same date as grip strength. The degree of grip fatigue (force‐decline rate) was calculated by comparing the first two and last two pulls. The formula (5th + 6th) / (1st + 2nd) * 100 (%) gives a measure of fatigue.

### Rotarod running test

Wild‐type, *mdx*, *mdx52* and *DMD‐null* (*n* = 7 for each group) mice at 3 months of age were tested on an accelerating rotarod apparatus (Ugo Basile, Comerio, Italy) set to accelerate from 5 to 45 rpm for 500 s.^35^ Time taken for each mouse to either fall off the rod or cling onto it for one complete rotation was recorded. Mice were tested for two more consecutive days, two trials per day. Maximum running time was used for further analysis.

### Western blotting

Total protein was extracted from tibialis anterior (TA) muscle and cerebral cortex, including motor area, using SDS buffer with protease inhibitors (Roche, Indianapolis, IN, USA). Lysates were crushed with beads and centrifuged at 14 000 × *g* for 15 min at 4°C. Supernatant was collected and protein concentrations were determined using a BCA protein assay kit (Thermo Fisher Scientific). After mixing with NuPAGE LDS Sample Buffer (Thermo Fisher Scientific), cell lysates were denatured at 70°C for 10 min, electrophoresed using NuPAGE Novex Tris‐Acetate Gel 3–8% (Invitrogen) at 150 V for 75 min, then transferred to PVDF membranes. Following three washes in PBS‐T, membranes were incubated with ECL western blot substrate (GE Healthcare Life Sciences). Membranes were incubated with primary antibodies, followed by incubation with a secondary antibody. Primary antibodies used were: rabbit anti‐dystrophin (ab15277, Abcam, 1/250; P34a from NCNP, 1/2000^36^), mouse anti‐ dystrophin (MAB1692, Minneapolis, 1/50; NCL‐DYSA, Leica, 1/250), and mouse anti‐GAPDH (MAB374, Sigma‐Aldrich, 1/100) antibody overnight at 4°C. Secondary antibodies (1/5000) of anti‐mouse or anti‐rabbit IgG horseradish peroxidase (HRP) linked F (ab′)2 fragment (Cytiva) for 60 min at room temperature. Data were analysed with Image Lab 6.0 (Bio‐Rad).

### Immunohistochemistry

Dystrophin‐positive revertant fibres were detected by immunohistochemistry. Transverse frozen sections (10 μm thick) from TA muscle were cut using a CryoStar NX70 cryostat (Thermo Fisher Scientific). Serial sections were picked up on poly‐l‐lysine‐coated glass microscope slides and air‐dried for 30 min. Unfixed sections were treated with 0.1% TritonX‐100 for 10 min then blocked in phosphate‐buffered saline (PBS) with 10% bovine serum albumin (BSA) for 1 h at room temperature. Dystrophin was detected with rabbit polyclonal primary antibody against human dystrophin C‐terminal (1/400) in the blocking solution by overnight incubation at 4°C. After three washes with PBS, primary antibody was detected with AlexaFluorTM 488‐conjugated goat anti‐rabbit IgG secondary antibody (1/2000) (Molecular Probes, OR, USA) with 1 h room temperature incubation. Nuclear counterstaining was performed with 4′,6‐diamidino‐2‐phenylindole (DAPI) in a mounting agent (Vectashield; Vector Laboratories, CA, USA). Muscle revertant fibre assessment was performed as previously reported.^37^

## Results

### Duchenne muscular dystrophy boys

Results from the studies in DMD boys are reported below grouped by *DMD* mutation expected effects on dystrophin isoform expression as follows; Group 1 (Dp427 absent, Dp140/Dp71 present); Group 2 (Dp427/Dp140 absent, Dp71 present); and Group 3 (Dp427/Dp140/Dp71 absent).

#### Patient characteristics

Of the 387 DMD boys studied, 201 were in Group 1, 152 in Group 2 and 34 in Group 3, *Table* 1. Group 2 participants had a 6 month earlier mean age of diagnosis than those in Group 1 (*P* = 0.05, *Table* 1).

**Table 1:** Patient characteristics

Demographics					
	Group 1 (*n* = 201)	Group 2 (*n* = 152)	Group 3 (*n* = 34)	All patients (*n* = 387)	*P* value between groups*
Mean (*SD*) age at diagnosis of DMD (years) G1 *n* = 171, G2 *n* = 123, G3 *n* = 31	4.2 (2.0)	3.6 (2.0)	3.8 (2.0)	3.9 (2.0)	0.05
Mean (*SD*) age range at baseline (years)	4.0, 15.4	4.0, 12.0	4.0, 12.2	4.0, 15.4	
GC use					
Mean (*SD*) age at initiation (years) G1 = 94, G2 = 71, G3 = 12	5.8 (1.4)	5.5 (1.3)	6.0 (1.5)	5.7 (1.5)	0.22
GC use recorded at any time (*n* and %)	189 (94.0%)	147 (96.7%)	31 (91.2%)	367 (94.8%)	0.32
Majority GC regimen					
Daily (*n* and %)	94 (46.8%)	78 (51.3%)	13 (38.2%)	185 (47.8%)	0.09
Int/other (*n* and %)	85 (42.3%)	58 (38.2%)	12 (35.3%)	155 (40.1%)	
None (*n* and %)	22 (10.9%)	16 (10.5%)	9 (26.5%)	47 (12.1%)	
Ambulatory function at 5 years of age					
	Group 1 (*n* = 44) Mean (*SD*)	Group 2 (*n* = 37) Mean (*SD*)	Group 3 (*n* = 9) Mean (*SD*)	All patients (*n* = 90)^a^ Mean (*SD*)	*P* value between groups*
NSAA score at 5 years of age	22.6 (5.4)	21.4 (5.0)	16.5 (6.7)	21.5 (5.6)	0.01
Rise from supine time at 5 years of age (seconds) G1 *n* = 40, G2 n = 37, G3 n = 9	4.9 (2.1)	4.9 (2.0)	9.5 (11.5)	5.4 (4.3)	<0.01
10 m walk/run time at 5 years of age (seconds) G1 *n* = 41, G2 *n* = 29, G3 n = 9	6.7 (2.6)	6.6 (2.4)	7.0 (2.2)	6.7 (2.5)	0.90
Loss of ambulation					
	Group 1 (*n* = 148)	Group 2 (*n* = 108)	Group 3 (*n* = 23)	All patients (*n* = 279)*	*P* value between groups*
Median (IQR) age of loss of ambulation (years)	15.7 (11.7, na)	13.0 (12.1, 14.9)	13.6 (12.9, 14.1)	13.6 (11.8, 16.1)	*P* = 0.67

DMD, Duchenne muscular dystrophy; G1, Group 1; G2, Group 2; G3, Group 3; GC, glucocorticoid; int, intermittent; na, not estimable; NSAA, North Star ambulatory assessment; *SD*, standard deviation.

^a^ Ninety patients had a NSAA recorded between ages 4.5–5.5 years.

* *P* values in this table represent *P* values for differences in the outcome when compared with isoform group as a whole. *P* values representing pairwise comparisons of isoform groups are quoted in the text.

Dystrophin isoform grouping in the above table is according to *DMD* mutation expected effects on dystrophin isoform expression as follows; Group 1 (Dp427 absent, Dp140/Dp71 present); Group 2 (Dp427/Dp140 absent, Dp71 present); and Group 3 (Dp427/Dp140/Dp71 absent).

We did not find a significant difference in age of GC initiation, GC regime or GC use recorded at any time between isoform groups (*Table* 1). There was a non‐significant trend for Group 3 to be more likely on no GC (*P* = 0.09, *Table* 1).

#### Motor function at 5 years of age

Mean NSAA scores at 5 years were over 6 points lower in Group 3 than Group 1 (*P* < 0.01) and 4.9 points lower in Group 3 than Group 2 (*P* = 0.05), with no difference between Groups 1 and 2 (*P* = 0.99, *Table* 1). Mean rise from supine times at 5 years were 4.6 s slower in Group 3 than Group 1 (*P* < 0.01) and 4.6 s slower in Group 3 than in Group 2 (*P* = 0.01), with no difference between Groups 1 and 2 (*P* = 0.99). There was no difference in mean 10MWR times at 5 years between isoform groups (*P* = 0.99).

#### Peak motor function

**Figure 1:**
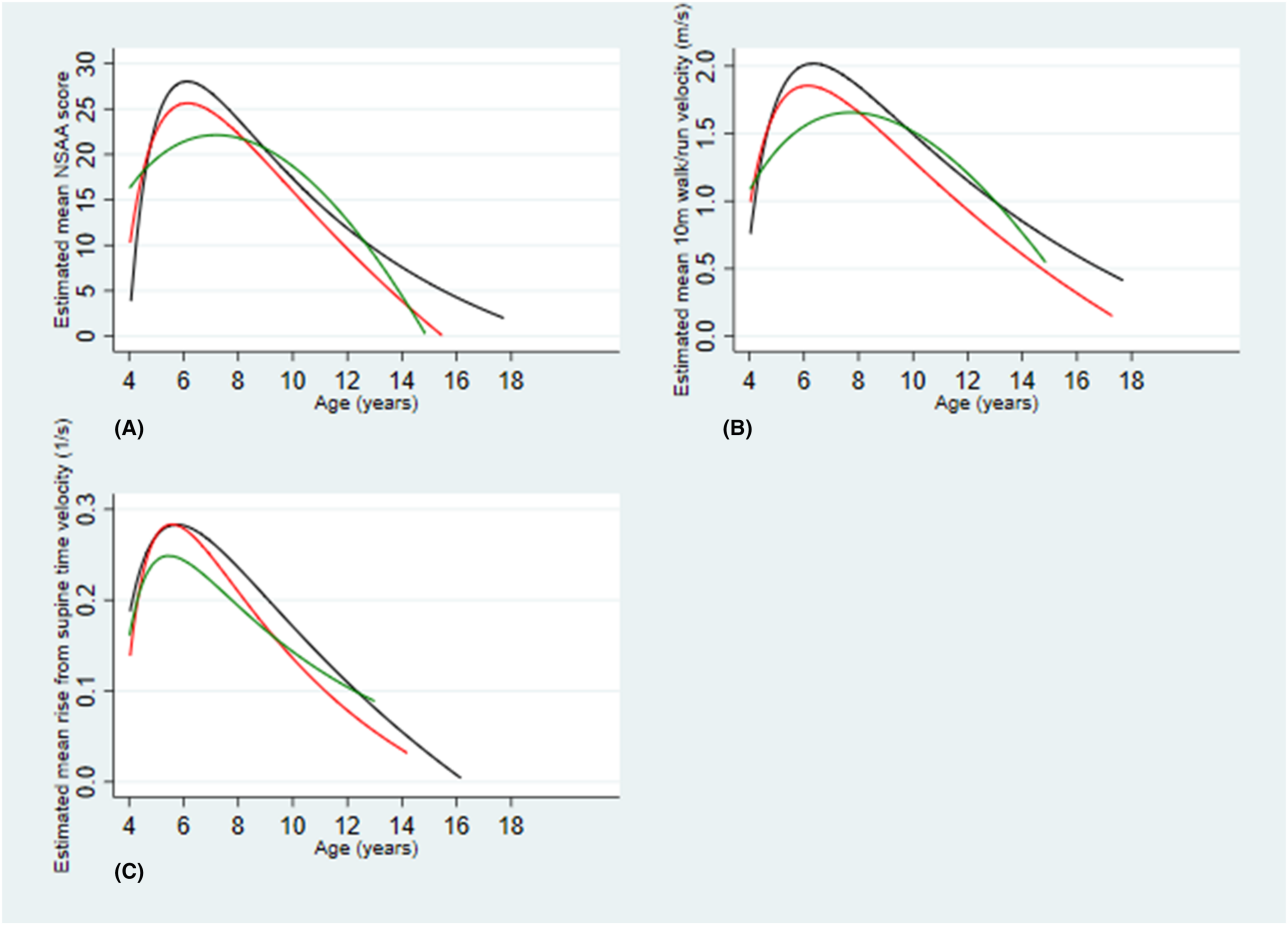
Estimated mean motor outcome trajectory with age models in the dystrophin isoform groups in Duchenne muscular dystrophy (DMD) boys. Dystrophin isoform grouping is according to *DMD* mutation expected effects on dystrophin isoform expression as follows; Group 1 (Dp427 absent, Dp140/Dp71 present); Group 2 (Dp427/Dp140 absent, Dp71 present); and Group 3 (Dp427/Dp140/Dp71 absent). Each line represents estimated mean motor outcome plotted against age for the dystrophin isoform group. Group 1 = blue, Group 2 = red, Group 3 = green. (A) represents estimated mean NSAA score trajectories with age, (B) represents estimated mean 10MWR velocities with age and (C) represents estimated mean rise from supine time velocities with age. 10 m = ten metre

Mean NSAA score trajectories peaked between 5.5 and 8.0 years (*Figure* 1). Therefore, we assessed differences in peak mean NSAA score, 10MWR velocity and rise from supine time velocity in the observed data for those aged 5.5–8.0 years (*Table* 2).

**Table 2:** Mean peak NSAA scores, mean peak 10 m walk/run velocities and mean peak rise from supine time velocities stratified by dystrophin isoform group after adjusting for GC regime

	Group 1	Group 2	Group 3	All groups
Mean (*SD*) peak NSAA score	26.6 (5.5)	25.0 (5.9)	22.1 (7.1)	25.6 (5.9)
	*n* = 129	*n* = 114	*n* = 20	*n* = 263
Mean (*SD*) peak 10 m walk/run velocity overall (m/s)	2.1 (0.5)	1.9 (0.6)	1.7 (0.5)	2.0 (0.6)
	*n* = 121	*n* = 108	*n* = 18	*n* = 247
Mean (*SD*) peak rise from supine time velocity overall (rises/s)	0.29 (0.12)	0.28 (0.13)	0.25 (0.12)	0.28 (0.12)
	*n* = 126	*n* = 105	*n* = 17	*n* = 248

GC, glucocorticoid; G1, Group 1; G2, Group 2; G3, Group 3.

This table considers the dataset for a subset (*n* = 263) of boys, aged 5.5–8.0 years, for whom GC regime data and peak North Star ambulatory assessment (NSAA)scores were available for all 262 boys. This subset of 5.5–8.0 years of age was used as this is the age in which the majority of participants reach peak motor function.^20^ Dystrophin isoform grouping reported in the above table is according to *DMD* mutation expected effects on dystrophin isoform expression as follows; Group 1 (Dp427 absent, Dp140/Dp71 present); Group 2 (Dp427/Dp140 absent, Dp71 present); and Group 3 (Dp427/Dp140/Dp71 absent). m/s = metres/second. rises/s = rises/second.

Mean peak NSAA scores were 4.0 points lower in Group 3 than Group 1 (*P* < 0.01), 2.4 points lower in Group 3 than Group 2 (*P* = 0.09) and 1.6 points lower in Group 2 than Group 1 (*P* = 0.04, *Table* 2).

Mean peak 10MWR velocity was 0.4 m/s [95% confidence interval (CI) 0.2–0.7] slower in Group 3 than Group 1 (*P* < 0.001) and 0.2 m/s (95% CI (0.05–0.4) slower in Group 2 than Group 1 (*P* < 0.001, *Table* 2), and non‐significantly lower in Group 3 compared with Group 2 (*P* = 0.10, *Table* 2).

Ages of mean peak NSAA score and 10MWR velocity were not significantly different between isoform groups (*Table* 2).

Mean peak rise from supine time velocity was 0.04 rises/s slower in Group 3 than Group 1 and 0.03 rises/s slower in Group 3 than Group 2; however, this was not significant (*Table* 2).

#### Age at loss of ambulation

Median age of LOA was lower in Groups 2 and 3 than Group 1; however, this did not reach statistical significance (*Table* 1).

#### Trajectory models

In NSAA score trajectory with age models, mean NSAA scores were lower in Group 2 than Group 1 and between 4.5 and 8.5 years of age were lower in Group 3 than in Groups 1 and 2 with cumulative effect of loss of isoforms (*Figure* 1). In 10MWR velocity trajectory with age models, velocities were higher in Group 1 than Groups 2 and 3 with a cumulative effect of loss of isoforms (*Figure* 1). In mean rise from supine time velocity trajectory with age models, Group 3 had a slower mean rise from supine time velocity than Groups 1 and 2 between approximately 5–11 years of age (*Figure* 1).

#### Relationship between isoform group and cognition group

In keeping with previous studies, DMD boys in isoform Groups 2 and 3 were more likely to be in the cognition impaired group than those in Group 1, with a cumulative effect of loss of isoforms (*P* < 0.001, *Table* S2).^12^, ^16^, ^17^, ^18^ 32% (20/63) of boys in Group 1 were in the impaired cognition group, compared with 53% (29/55) in Group 2 and 78% (7/9) in Group 3 (*Table* S2).

#### Relationships between cognition group and NSAA scores

We evaluated the influence of cognitive impairment on peak NSAA scores in a subset of 127 boys for whom cognition group, GC regimen and peak NSAA scores were available. Mean peak NSAA scores were 2.2 points lower in those in the impaired cognition group than those in the normal cognition group (*P* = 0.04, *Table* S2).

In NSAA trajectory models stratified by cognition group, mean NSAA scores were lower in the impaired cognition group than those in the normal cognition group (*Figure* S1).

### Human pathology studies

Results from the human pathology studies are reported below grouped by *DMD* mutation expected effects on dystrophin isoform expression as follows; Group 1 (Dp427 absent, Dp140/Dp71 present); Group 2 (Dp427/Dp140 absent, Dp71 present); and Group 3 (Dp427/Dp140/Dp71 absent).

**Figure 2:**
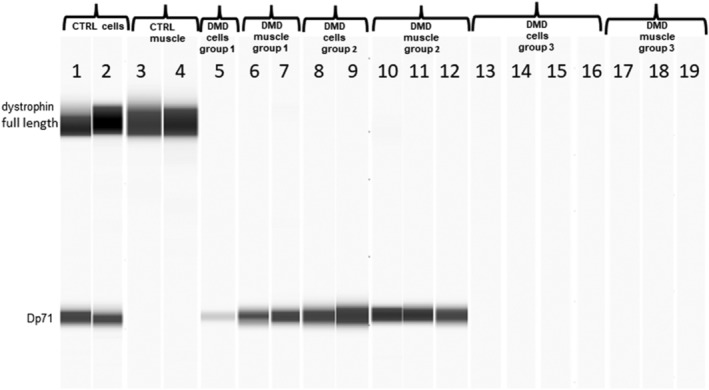
Virtual blot of dystrophin signal detected by Wes using C‐terminus polyclonal anti dystrophin antibodies (Abcam 15277 and 154168). Lanes:1 and 2) control fibroblasts MyoD transduced; 3 and 4) control muscles (CTRL); 5) Duchenne muscular dystrophy (DMD) myotubes Group 1; 6 and 7) DMD muscle Group 1; 8 and 9) DMD fibroblasts MyoD transfected Group 2; 10,11 and 12) DMD muscle Group 2; 13, 14, 15 and 16) DMD cells Group 3; 17,18 and 19 DMD muscle Group 3. All the patient details are summarized in *Table* S1. Full length dystrophin bands can only be observed in control cells and muscles. Patients with *DMD* mutations not expected to disrupt Dp71 production showed the Dp71 signal (Groups 1 and 2). No Dp71 bands in Group 3 patients can be detected. Dystrophin isoform grouping is according to *DMD* mutation expected effects on dystrophin isoform expression as follows; Group 1 (Dp427 absent, Dp140/Dp71 present); Group 2 (Dp427/Dp140 absent, Dp71 present); and Group 3 (Dp427/Dp140/Dp71 absent).

In the Wes studies, a band corresponding to Dp427 was seen in control myogenic cells and muscle, but not DMD cells or DMD muscle (*Figure* 2). Dp140 expression could not be detected in any samples studied. A band corresponding to Dp71 was seen in control and DMD myogenic cells and muscle from isoform Groups 1 and 2, but not DMD myogenic cells and muscle from patients in isoform Group 3 (*Figure* 2).

#### Immunoperoxidase staining of muscle sections

Sarcolemmal staining to C‐terminus dystrophin (Dys2, Novocastra) was present in all control muscle fibres, but no staining was seen in sections from any boys with DMD in dystrophin isoform Groups 1–3, apart from a single fibre in one section from one patient in Group 1 (*Figure* S2).

### Mouse studies

**Figure 3:**
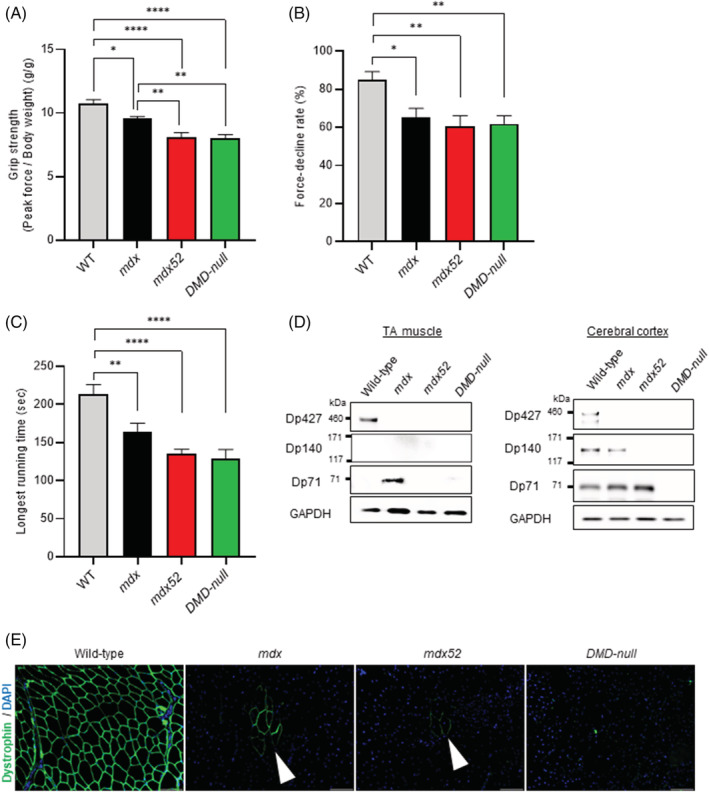
Muscle function and dystrophin expression analysis in wild‐type, *mdx* (Dp427 absent, Dp140/Dp71 present), *mdx52* (Dp427/Dp140 absent, Dp71 present) and *DMD‐null* (lacking all dystrophin isoforms) mice at the age of 3 months. The data are presented as mean ± SEM. Statistical differences were assessed by one‐way analysis of variance with differences among the groups assessed by a Dunnett comparison. (A) Repeat grip strength testing. *P* = 0.0166 (wild‐type vs. *mdx*), *P* < 0.0001 (wild‐type vs. *mdx52*), *P* < 0.0001 (wild‐type vs. *DMD‐null*), *P =* 0.0032 (*mdx* vs. *mdx52*), *P =* 0.0023 (*mdx* vs. *DMD‐null*). *N* = 10 (wild‐type), *n* = 10 (*mdx*), *n* = 10 (*mdx52*) and *n* = 10 (*DMD‐null*) (B) force decline rate. *P* = 0.0139 (wild‐type vs *mdx*), *P* = 0.0021 (wild‐type vs *mdx52*), *P* = 0.0039 (wild‐type vs *DMD‐null*). *n* = 10 (wild‐type), *n* = 10 (*mdx*), *n* = 10 (mdx52) and *n* = 10 (*DMD‐null*) (C) Rotarod running test. *P* = 0.0088 (wild‐type vs. *mdx*), *P* < 0.0001 (wild‐type vs. *mdx52*), *P* < 0.0001 (wild‐type vs. *DMD‐null*). *n* = 7 (wild‐type), *n* = 7 (*mdx*), *n* = 7 (*mdx52*) and *n* = 7 (*DMD‐null*) (D) dystrophin western blot using anti dystrophin antibodies (Abcam 15277, NCL‐DYSA, MAB1692, P34a) and anti‐GAPDH antibodies (as an internal standard) in tibialis anterior (TA) muscle and cerebral cortex, including motor area. *n* = 1 (wild‐type), *n* = 1 (*mdx*), *n* = 1 (*mdx52*) and *n* = 1 (*DMD‐null*) (E) dystrophin immunochemistry using a C‐terminus polyclonal anti dystrophin antibody (Abcam 15277) in TA muscle. White arrowheads denote revertant fibres. *P < 0.05; **P < 0.01; ***P < 0.001; ****P < 0.0001.

Mouse study results are reported for 3 DMD mouse models; *mdx* (Dp427 absent, Dp140/Dp71 present), *mdx52* (Dp427/Dp140 absent, Dp71 present) and *DMD‐null* (lacking all dystrophin isoforms).^31^, ^32^ Average four‐limb grip strength in peak force at 3 months of age was 1.5 g/g lower in *mdx52* than *mdx* mice (*P* = 0.003) and 1.5 g/g lower in *DMD‐null* than *mdx* mice (*P* = 0.002), with no statistically significant difference between *mdx52* and *DMD‐null mice* (*Figure* 3A and *Table* S3). There were no statistically significant differences in grip fatigue and longest‐running time between the DMD mouse models (*Figure* 3B,C and *Table* S3). In TA muscle, Dp427 was detected in wild‐type mice and not the DMD mouse models; Dp140 was not detected in any TA muscle studied; and Dp71 was detected in *mdx*, but not wild‐type, *mdx52* or *DMD‐null* mice by dystrophin western blot using DysA antibody (*Figure* 3D). In cerebral cortex near the motor area, Dp140 was detected in wild‐type and *mdx* mice, but not *mdx52* or *DMD‐null* mice, and Dp71 was detected in wild‐type, *mdx* and *mdx52* mice, but not *DMD‐null* mice, by western blot using P34a antibody (*Figure* 3D). Revertant fibres were detected in TA muscle in *mdx* (32 revertant fibres/section) and *mdx52* (9 revertant fibres/section) mice, but not *DMD‐null* mice (*Figure* 3E).

## Discussion

We hypothesized that *DMD* mutations expected to differentially impact on dystrophin isoform production could differentially affect motor function in DMD in humans and mouse models. We stratified motor outcome data from boys with DMD based on the predicted effect of their *DMD* mutations on three dystrophin isoforms produced in the brain (Dp427, Dp140 and Dp71) and cognitive function. We evaluated production of these three isoforms in normal and DMD myogenic and skeletal muscle from patient's representative of the three isoform groups. Finally, we characterized functional abilities and assessed dystrophin isoform production in muscle and brain in wild‐type and three DMD mouse models with differential production of the three isoforms.

Our results from DMD boys clearly demonstrate marked reductions in motor function at 5 years of age (reduced mean NSAA score and rise from supine time) and peak motor function (reduced mean peak NSAA score and 10MWR velocity) in those lacking Dp140 and Dp71, with a clear cumulative effect of loss of isoforms. Mean peak NSAA score was lower in those with cognitive impairment than those with normal cognition. Differences in NSAA score between isoform groups were considerable, often exceeding the minimally clinically important difference of approximately three points.^38^ It is not current practice to stratify clinical trial patients according to expected dystrophin isoform involvement. Clinical trials are increasingly recruiting younger DMD boys aged within the key time points of our study.^39^ ^–42^ NSAA scores, rise from supine times and 10MWR times are frequently used as DMD clinical trial outcome measures.^39^ ^–42^ Not considering expected effects of *DMD* mutation type on dystrophin isoform production when recruiting patients to treatment and placebo arms could result in an imbalance of background DMD severity that clouds interpretation of clinical trial results and/or masks treatment effects.

We demonstrated that grip strength in *mdx52* and *DMD‐null* mice was significantly reduced compared with *mdx* mice. However, we did not detect a significant difference in grip strength between *DMD‐null* and *mdx52* mice. This suggests a strong association between lack of Dp140 and poorer grip strength in DMD mouse models. A recent study identified significant reduction in locomotion of *mdx52* compared with *mdx* mice, further supporting our findings.^43^ While the lack of additional functional impairment in the *DMD‐null* compared with the *mdx52* is at odds with our clinical findings in the boys expected to be Dp71 deficient, it is interesting to note that deficiency of all DMD isoforms in the *mdx3cv* mouse is not associated with a more severe learning impairment compared with *mdx*,^44^ in sharp contrast with the major effect of Dp71 deficiency on human cognitive function.^12^, ^13^, ^14^, ^15^, ^16^

Our human and mouse data suggest differences in outcomes between isoform groups are not likely related to an effect of these isoforms on the severity of dystrophic muscle pathology.

We firstly considered whether differences in Dp140 and Dp71 production in skeletal muscle could have determined these functional outcomes, but we discarded this hypothesis.

Regarding Dp140, several studies indicate Dp140 expression is restricted to the brain and developing kidney.^8^, ^11^ ^,45,46^ Our study confirms Dp140 absence in all muscle and myogenic cultures studied. As expected, Dp140 deficiency was observed in *mdx52* and *DMD‐null* brain, while Dp140 production was preserved in *mdx* and wild‐type mouse cortex. We cannot exclude a possible compromised role of Dp140 in motoneuron function contributing to the worse trajectories in Dp140 deficient boys and mice.^43^ Future neuropathological motoneuron studies with mutations affecting Dp140 would be required to address this. Nevertheless, a major role of Dp140 deficiency on cognitive function has been universally recognized in DMD humans and mouse models.^16^, ^17^, ^18^ ^,43,47^

Regarding Dp71, Dp71 is expressed more ubiquitously, with transcripts detectable in brain and also skeletal muscle, during fetal development and adult life.^48,49^ The Dp71 promoter is ubiquitous, and Dp71 is transcribed at similar levels during development and adult life. However, higher protein levels during development suggest a significant degree of Dp71 post‐transcriptional regulation.^48^ In our studies, Dp71 was produced in small amounts in control and DMD myogenic cells, muscle from DMD boys in isoform Groups 1 and 2 and *mdx* mouse muscle, but not detected in muscle from wild‐type, *mdx52* and *DMD‐null* mice or DMD patients in isoform Group 3.

Despite the low level detection of Dp71 in muscle in DMD boys and *mdx* mice using Wes, endogenous Dp71 cannot be visualized at the sarcolemma.^49,50^ We also could not detect sarcolemmal dystrophin staining in any DMD human muscle studied from the three isoform groups, in keeping with the notion that DMD patients and *mdx* mice demonstrate absent C‐terminal dystrophin at the sarcolemma.^30^ ^,49,50^ A recent study suggested a tagged Dp71 is expressed in nucleoplasm.^51^ Sarcolemmal localization of Dp71 can, however, be detected by transgenic experimental studies overexpressing Dp71 in myogenic cell lines^52^ or on a wild‐type background, where Dp71 overexpression is associated with muscle degeneration resembling muscular dystrophy.^53^ Dp71 overexpression in *mdx* mouse muscle is unable to avoid progressive muscle pathology despite partially increasing expression of proteins of the dystrophin associated glycoprotein complex (DAPC).^54,55^ This likely relates to the fact that while Dp71 binds to the DAPC protein beta dystroglycan, it lacks the Dp427 actin‐binding domain, which is essential for protecting muscle from contraction‐induced damage.

The higher level of Dp71 observed during muscle development and its role during early myogenesis might provide an explanation for Dp71 detection by Wes in DMD and *mdx* mouse muscle.^56^ Several studies have suggested a role for Dp71 in myoblast cell proliferation and satellite cell activation,^57–59^ suggesting that low‐level Dp71 production in DMD muscle is a secondary phenomenon related to the muscle degeneration and regeneration characteristic of this condition.

Finally, we considered the possible role of different amounts of revertant fibres between mouse models. We detected revertant fibres at higher levels in TA muscle in *mdx* than *mdx52* mice, but not in *DMD‐null* mice, in keeping with previous findings.^60^ While we cannot exclude a small contribution of this finding to the worse outcome of *mdx52*, lack of further decrease in grip strength in the *DMD‐null*, completely lacking revertant fibres, argues against this.

Considering the role of Dp140 and Dp71 in brain function, a plausible explanation for our findings is that the observed differences in motor function could be related to a direct impact of CNS involvement on motor executive function.^12^, ^17^, ^18^ ^,61^ While the concept of an impact of brain function on motor performance is novel in the DMD field, this is well‐recognized in other conditions. Children with learning disability have been found to have lower locomotor scores than typically developing children.^62^

Duchenne muscular dystrophy boys exhibit executive function deficits, specifically planning and directing goal oriented behaviour.^63^ Dp140 negative male participants exhibit slower information processing speeds than Dp140 positive.^64^ *Mdx52* mice exhibit more severe amygdala dependent Pavlovian learning impairment than *mdx* mice.^43^

Previous studies have hypothesized a role of the cerebellum and/or the cerebellar thalamic cortical connectivity in DMD cognitive difficulties.^63^ Dp427, Dp140 and Dp71 are all expressed in adult human cerebellum.^8^ The cerebellum plays a crucial role in control of goal‐directed movements and timing of coordinated movement.^65^ Features of paediatric cerebellar movement dysfunction include gross motor delay and poor coordination with complex movements (with relative sparing of simple motor tasks).^66^ Of NSAA, rise from supine velocity and 10MWR velocity, NSAA score was the only outcome showing impairment in those expected to lack Dp140 and Dp140/Dp71 at both 5 years and peak motor function. Of these three outcomes, NSAA is the most complex, requiring the highest level of motor coordination and planning.

Taken together, we consider the most plausible explanation of our findings is that the more severe motor impairment in boys and *mdx52* mice lacking Dp140 or Dp140/Dp71 are linked to deficits in higher centres of motor control and coordination, with a possible role of deficits in the cerebellum and/or the cerebellar thalamic cortical connectivity.

The strengths of this study include the large patient numbers, ‘real world’ clinical setting of data collection and the longitudinal data. Limitations included some missing data, typical of a real‐world setting, which we nevertheless accounted for statistically.

In summary, we found that *DMD* mutations expected to lead to deficiency of dystrophin isoforms produced in the brain are associated with poorer motor function in DMD boys and mouse models, with a cumulative effect of loss of isoforms in DMD boys. Given these isoforms' major role in brain function, we hypothesize this could be at least partly related to deficits in higher centres of motor control and coordination in those lacking Dp140 and Dp140/Dp71.

Irrespective of the pathophysiological explanation, our novel findings provide evidence for a relationship between *DMD* mutation site and its effect on Dp140 and Dp71 production on not only cognitive, but also motor outcomes, with crucial implications for clinical practice and clinical trial design.

## Conflicts of interest

- Mary Chesshyre, Deborah Ridout, Kate Maresh, Lianne Abbott, Vandana Ayyar Gupta, Marion Main, Yoko Ookubo, Yasumasa Hashimoto, Yoshitsugu Aoki, Silvia Torelli, Giulia Ferrari and Anna Kowala report no financial disclosures or potential conflicts of interest.
- Valeria Ricotti—Dr Valeria Ricotti is co‐founder, EVP and CMO of DiNAQOR and shareholder of Solid Biosciences
- Adnan Manzur—Dr Adnan Manzur is the clinical lead for the North Star clinical network and is one of the co‐ grant holders from the MDUK for maintenance of the network.
- Yung‐Yao Lin—Dr Yung‐Yao Lin has received research funding from Pfizer.
- Francesco Saverio Tedesco – Professor Francesco Saverio Tedesco has received speaker and consultancy honoraria from Takeda, Sanofi Genzyme and Aleph Farms (via UCL Consultants)
- Mariacristina Scoto—Dr Mariacristina Scoto has received speaker and consultancy honoraria For Roche, Avexis, Santhera and Biogen
- Giovanni Baranello—Dr Giovanni Baranello has received speaker and consultancy honoraria from AveXis, Roche, PTC, and Sarepta Therapeutics
- Francesco Muntoni—Professor Francesco Muntoni is supported by the NIHR Great Ormond Street Hospital Biomedical Research Centre and has received speaker and consultancy honoraria from Sarepta Therapeutics, Avexis, PTC Therapeutics, Roche and Pfizer.

## Ethical guidelines and consent

All authors certify that they comply with the ethical guidelines and publishing in the *Journal of Cachexia, Sarcopenia and Muscle*.^67^ Written informed consent was obtained for the collection of all clinical data and human tissue samples and the NorthStar clinical network project has Caldicott Guardian approval. All clinical assessments are conducted according to the principles of the Declaration of Helsinki (2000) and its later amendments and the Principles of Good Clinical Practice. Fibroblasts, iPSCs and muscle tissues were supplied by the MRC Centre for Neuromuscular Disease Biobank London (REC reference number 06/Q0406/33). The human pathology studies were conducted under the REC reference numbers 13/LO/1894 and 13/LO/1826. Animal studies were performed in accordance with the appropriate ethics committee and have therefore been performed with the ethical standards laid down in the 1964 Declaration of Helsinki and its later amendments.

## Supporting information


**Table S1.** Sample type, *DMD* mutation, location and isoform group for samples used in analysis for Figure 2.
**Table S2.** Relationships between dystrophin isoform group and cognition group and between cognition group and peak NSAA scores.
**Table S3.** Mean peak 4‐limb grip strength at 3 months of age, mean force decline rate and mean longest running time in rotarod running test for wild‐type (WT), *mdx*, *mdx52* and *DMD‐null* mice.

**Figure S1**. Estimated mean NSAA score trajectory with age models in those with and without cognitive impairment.
**Figure S2.** Immunoperoxidase staining of muscle sections.

Supplementary references (references 40 to 67).
