# Supplementary material for: Investigating the role of dystrophin isoform deficiency in motor function in Duchenne muscular dystrophy
Source: J Cachexia Sarcopenia Muscle. 2022 Jan 26;13(2):1360–72. doi: 10.1002/jcsm.12914 (PMC8977977; doi:10.1002/jcsm.12914)
Supplement: Supplementary file 1 — Table S1. Sample type, DMD mutation, location and isoform group for samples used in analysis for Figure 2. Table S2. Relationships between dystrophin isoform group and cognition group and between cognition group and peak NSAA scores. Table S3. Mean peak 4‐limb grip strength at 3 months of age, mean force decline rate and mean longest running time in rotarod running test for wild‐type (WT), mdx, mdx52 and DMD‐null mice. [file JCSM-13-1360-s002.pdf]

Investigating the Role of Dystrophin Isoform Deficiency in Motor Function in Duchenne Muscular Dystrophy

Mary Chesshyre[1,3], Deborah Ridout[2,3], Yasumasa Hashimoto[4], Yoko Ookubo[4], Silvia Torelli[1], Kate Maresh[1,3], Valeria Ricotti[1,3,] Lianne Abbott[1,3], Vandana Ayyar Gupta[1], Marion Main[1,3], Giulia Ferrari[5], Anna Kowala[6], Yung-Yao Lin[6], Francesco Saverio Tedesco[1,5,7], Mariacristina Scoto[1,3], Giovanni Baranello[1,3], Adnan Manzur[1,3], Yoshitsugu Aoki[4] and Francesco Muntoni[1, 3]

On behalf of the North Star clinical network

1. Dubowitz Neuromuscular Centre, UCL Great Ormond Street Institute of Child Health, 30 Guilford Street, London, WC1N 1EH, United Kingdom
2. Population, Policy and Practice Research and Teaching Department, UCL Great Ormond Street Institute of Child Health, 30 Guilford Street, London, WC1N 1EH, United Kingdom
3. NIHR Great Ormond Street Hospital Biomedical Research Centre, UCL Great Ormond Street Institute of Child Health, 30 Guilford Street, London, WC1N 1EH, United Kingdom
4. Department of Molecular Therapy, National Institute of Neuroscience, National Center of Neurology and Psychiatry (NCNP), Kodaira, Tokyo 187-8502, Japan
5. Department of Cell and Developmental Biology, University College London, London, WC1E 6DE, United Kingdom
6. Centre for Genomics and Child Health, Blizard Institute, Barts and the London School of Medicine and Dentistry, Queen Mary University of London, 4 Newark Street, London E1 2AT, United Kingdom
7. The Francis Crick Institute, 1 Midland Road, London, NW1 1AT, United Kingdom

Corresponding author
Professor Francesco Muntoni
Dubowitz Neuromuscular Centre
UCL Great Ormond Street Institute of Child Health
30 Guilford Street
London
WC1N 1EH
United Kingdom
f.muntoni@ucl.ac.uk

Journal of Cachexia, Sarcopenia and Muscle – Supplementary material. Table S1, Table S2 and Table S3

Supplementary material

Table S1. Sample type, *DMD* mutation, location and isoform group for samples used in analysis for Fig. 2

Sample	*DMD* mutation	Location	Isoform group
1. Control fibroblasts MyoD transfected	N/A	Skin	CTRL
2. Control fibroblasts MyoD transfected	N/A	Skin	CTRL
3. CTRL muscle	N/A	Quad	CTRL
4. CTRL muscle	N/A	Quad	CTRL
5. DMD myogenic cells	Point mutation exon 12	Quad	Group 1
6. DMD muscle	Point mutation exon 32	Quad	Group 1
7. DMD muscle	Deletion exons 10-11	Quad	Group 1
8. DMD fibroblasts MyoD transfected	Deletion exon 52	Skin	Group 2
9. DMD fibroblasts MyoD transfected	Deletion exon 52	Skin	Group 2
10. DMD muscle	Deletion exons 48-54	Quad	Group 2
11. DMD muscle	Point mutation exon 52	Quad	Group 2
12. DMD muscle	Deletion exon 52	Quad	Group 2
13. DMD myogenic iPSc	Point mutation exon 71	Skin	Group 3
14. DMD myogenic iPSc	Point mutation exon 68	Skin	Group 3
15. DMD myogenic cells	Point mutation exon 70	Skin	Group 3
16. DMD myogenic cells	Point mutation exon 73	Skin	Group 3
17. DMD muscle	Point mutation exon 67	EDB	Group 3
18. DMD muscle	Point mutation exon 70	Quad	Group 3
19. DMD muscle	Point mutation exon 70	Quad	Group 3

Table S1. Sample type, *DMD* mutation, location and isoform group for samples used in analysis for Fig. 2. CTRL= control, Quad = quadriceps, EDB = extensor digitorum brevis. Dystrophin isoform grouping is according to *DMD* mutation expected effects on dystrophin isoform expression as follows; Group 1 (Dp427 absent, Dp140/Dp71 present); Group 2 (Dp427/Dp140 absent, Dp71 present); and Group 3 (Dp427/Dp140/Dp71 absent).

Table S2. Relationships between dystrophin isoform group and cognition group and between cognition group and peak NSAA scores

Relationships between dystrophin isoform group and cognition group			
Dystrophin isoform group	Normal cognition	Impaired cognition	
Group 1 (n, %)	43 (68%)	20 (32%)	
Group 2 (n, %)	26 (47%)	29 (53%)	
Group 3 (n, %)	2 (22%)	7 (78%)	
Age of diagnosis and starting GC stratified by cognition group			
Age of diagnosis in years<br>Mean (sd)	3.4 (1.7)	3.1 (1.7)	
Age of starting GC in years<br>Mean (sd)	5.1 (1.4)	5.2 (0.8)	
Peak NSAA scores stratified by cognition group			
	Normal cognition	Impaired cognition	Both groups combined
Mean (sd) peak NSAA score	27.7 (5.3)	25.3 (5.9)	26.6 (5.7)
Number in each group	71	56	127

Table S2. Relationships between dystrophin isoform group and cognition group and between cognition group and peak NSAA scores, after adjusting for GC regime. GC=glucocorticoid. This table considers the subset of participants (n=127) for whom cognition grouping, GC regimen and peak NSAA scores were available. Dystrophin isoform grouping is according to *DMD* mutation expected effects on dystrophin isoform expression as follows; Group 1 (Dp427 absent, Dp140/Dp71 present); Group 2 (Dp427/Dp140 absent, Dp71 present); and Group 3 (Dp427/Dp140/Dp71 absent).

<u>**Table S3.** Mean peak 4-limb grip strength at 3 months of age, mean force decline rate and mean longest running time in rotarod running test for wild-type (WT), *mdx*, *mdx52* and *DMD-null* mice.</u>

		Mean peak 4-limb grip strength at 3 months of age / Body weight (g/g)	Mean force decline rate (%)	Mean longest running time in rotarod running test (sec)
WT		10.7	85.2	213.9
mdx		9.5	65.1	164.7
mdx52		8.1	60.4	135.6
DMD-null		8.0	61.8	128.7
P values	WT vs *mdx*	0.017	0.014	0.009
	WT vs *mdx52*	<0.0001	0.002	<0.0001

	WT vs *DMD-null*	<0.0001	0.003	<0.0001
	mdx vs mdx52	0.003	0.824	0.155
	mdx vs DMD-null	0.002	0.932	0.063
	mdx52 vs DMD-null	0.999	0.993	0.940

Table S3. Mean peak 4-limb grip strength at 3 months of age, mean force decline rate and mean longest running time in rotarod running test for wild-type (WT), *mdx* (Dp427 absent, Dp140/Dp71 present), *mdx52* (Dp427/Dp140 absent, Dp71 present) and *DMD-null* (lacking all dystrophin isoforms) mice.
