# Supplementary material for: Investigating the role of dystrophin isoform deficiency in motor function in Duchenne muscular dystrophy
Source: J Cachexia Sarcopenia Muscle. 2022 Jan 26;13(2):1360–72. doi: 10.1002/jcsm.12914 (PMC8977977; doi:10.1002/jcsm.12914)
Supplement: Supplementary file 2 — Figure S1. Estimated mean NSAA score trajectory with age models in those with and without cognitive impairment. Figure S2. Immunoperoxidase staining of muscle sections. [file JCSM-13-1360-s001.pdf]

Investigating the Role of Dystrophin Isoform Deficiency in Motor Function in Duchenne Muscular Dystrophy

Mary Chesshyre[1,3], Deborah Ridout[2,3], Yasumasa Hashimoto[4], Yoko Ookubo[4], Silvia Torelli[1], Kate Maresh[1,3], Valeria Ricotti[1,3,] Lianne Abbott[1,3], Vandana Ayyar Gupta[1], Marion Main[1,3], Giulia Ferrari[5], Anna Kowala[6], Yung-Yao Lin[6], Francesco Saverio Tedesco[1,5,7], Mariacristina Scoto[1,3], Giovanni Baranello[1,3], Adnan Manzur[1,3], Yoshitsugu Aoki[4] and Francesco Muntoni[1, 3]

On behalf of the North Star clinical network

1. Dubowitz Neuromuscular Centre, UCL Great Ormond Street Institute of Child Health, 30 Guilford Street, London, WC1N 1EH, United Kingdom
2. Population, Policy and Practice Research and Teaching Department, UCL Great Ormond Street Institute of Child Health, 30 Guilford Street, London, WC1N 1EH, United Kingdom
3. NIHR Great Ormond Street Hospital Biomedical Research Centre, UCL Great Ormond Street Institute of Child Health, 30 Guilford Street, London, WC1N 1EH, United Kingdom
4. Department of Molecular Therapy, National Institute of Neuroscience, National Center of Neurology and Psychiatry (NCNP), Kodaira, Tokyo 187-8502, Japan
5. Department of Cell and Developmental Biology, University College London, London, WC1E 6DE, United Kingdom
6. Centre for Genomics and Child Health, Blizard Institute, Barts and the London School of Medicine and Dentistry, Queen Mary University of London, 4 Newark Street, London E1 2AT, United Kingdom
7. The Francis Crick Institute, 1 Midland Road, London, NW1 1AT, United Kingdom

Corresponding author

Professor Francesco Muntoni

Dubowitz Neuromuscular Centre

UCL Great Ormond Street Institute of Child Health

30 Guilford Street

London

WC1N 1EH

United Kingdom

f.muntoni@ucl.ac.uk

Journal of Cachexia, Sarcopenia and Muscle – Supplementary material. Figure S1 and Figure S2

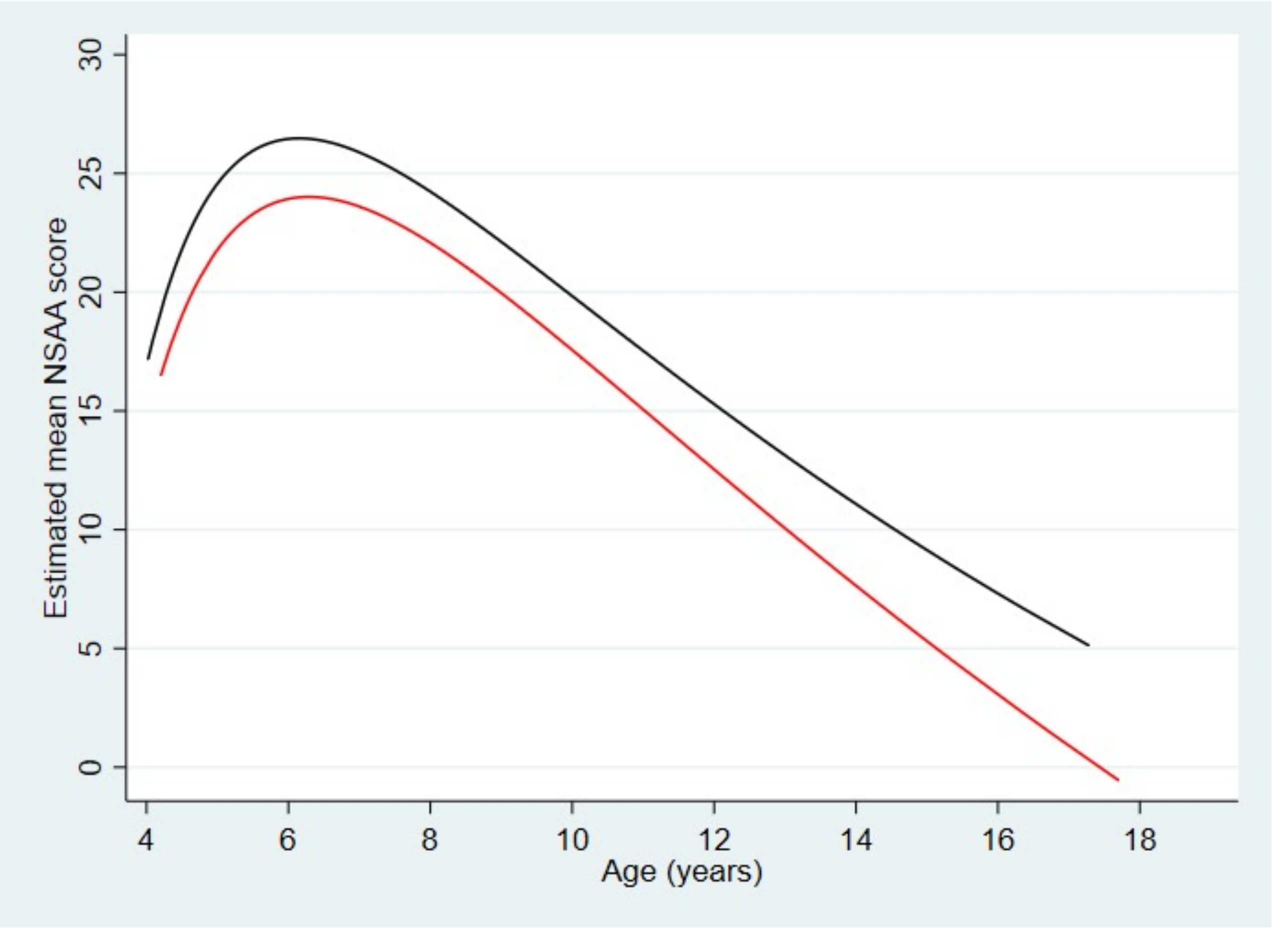

Figure S1. Estimated mean NSAA score trajectory with age models in those with and without cognitive impairment. Each line represents the estimated mean NSAA score trajectory plotted against age for the cognition group. Black = normal cognition and red = impaired cognition.

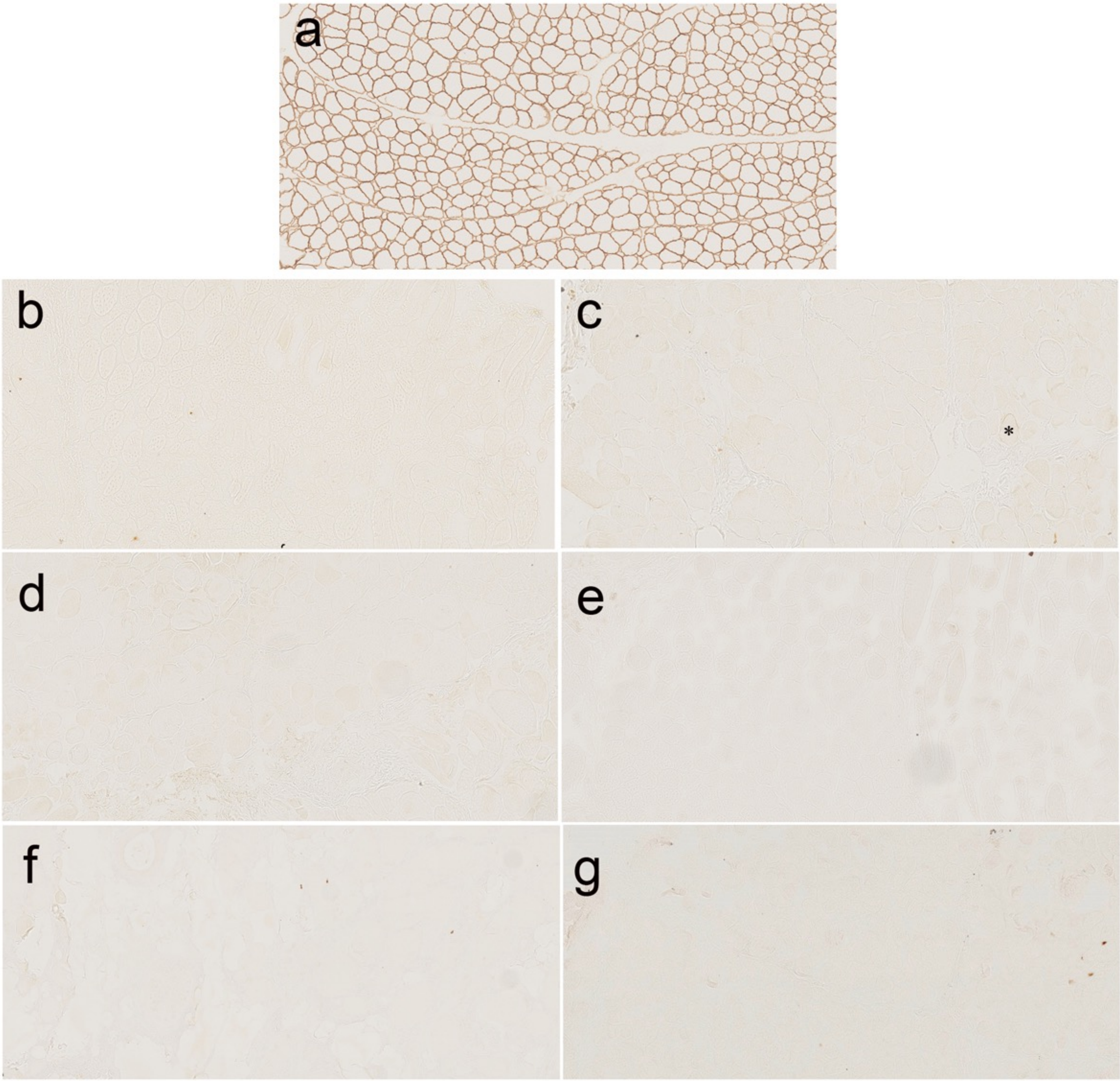

Figure S2. Immunoperoxidase staining of muscle sections. Representative images of patient skeletal muscle sections from each group, immunostained with antibody to C-terminus dystrophin (Dys2, Novocastra). a) control muscle; b) dystrophin isoform Group 1 ; c) dystrophin isoform Group 1; d) dystrophin isoform Group 2; e) dystrophin isoform Group 2; f) dystrophin isoform Group 3: g) dystrophin isoform Group 3. Sarcolemmal staining is present in all fibres in control muscle (a), while no staining can be seen in all the other sections (b-g) except one fibre in section c (marked with *). Dystrophin isoform grouping is according to *DMD* mutation expected effects on dystrophin isoform expression as follows; Group 1 (Dp427 absent, Dp140/Dp71 present); Group 2 (Dp427/Dp140 absent, Dp71 present); and Group 3 (Dp427/Dp140/Dp71 absent).
