# Supplementary material for: Investigating the role of dystrophin isoform deficiency in motor function in Duchenne muscular dystrophy
Source: J Cachexia Sarcopenia Muscle. 2022 Jan 26;13(2):1360–72. doi: 10.1002/jcsm.12914 (PMC8977977; doi:10.1002/jcsm.12914)
Supplement: Supplementary file 3 — Supplementary references (references 40 to 67). [file JCSM-13-1360-s003.pdf]

Investigating the Role of Dystrophin Isoform Deficiency in Motor Function in Duchenne Muscular Dystrophy

Mary Chesshyre[1,3], Deborah Ridout[2,3], Yasumasa Hashimoto[4], Yoko Ookubo[4], Silvia Torelli[1], Kate Maresh[1,3], Valeria Ricotti[1,3,] Lianne Abbott[1,3], Vandana Ayyar Gupta[1], Marion Main[1,3], Giulia Ferrari[5], Anna Kowala[6], Yung-Yao Lin[6], Francesco Saverio Tedesco[1,5,7], Mariacristina Scoto[1,3], Giovanni Baranello[1,3], Adnan Manzur[1,3], Yoshitsugu Aoki[4] and Francesco Muntoni[1, 3]

On behalf of the North Star clinical network

1. Dubowitz Neuromuscular Centre, UCL Great Ormond Street Institute of Child Health, 30 Guilford Street, London, WC1N 1EH, United Kingdom
2. Population, Policy and Practice Research and Teaching Department, UCL Great Ormond Street Institute of Child Health, 30 Guilford Street, London, WC1N 1EH, United Kingdom
3. NIHR Great Ormond Street Hospital Biomedical Research Centre, UCL Great Ormond Street Institute of Child Health, 30 Guilford Street, London, WC1N 1EH, United Kingdom
4. Department of Molecular Therapy, National Institute of Neuroscience, National Center of Neurology and Psychiatry (NCNP), Kodaira, Tokyo 187-8502, Japan
5. Department of Cell and Developmental Biology, University College London, London, WC1E 6DE, United Kingdom
6. Centre for Genomics and Child Health, Blizard Institute, Barts and the London School of Medicine and Dentistry, Queen Mary University of London, 4 Newark Street, London E1 2AT, United Kingdom
7. The Francis Crick Institute, 1 Midland Road, London, NW1 1AT, United Kingdom

Corresponding author
Professor Francesco Muntoni
Dubowitz Neuromuscular Centre
UCL Great Ormond Street Institute of Child Health
30 Guilford Street
London
WC1N 1EH
United Kingdom
f.muntoni@ucl.ac.uk

Journal of Cachexia, Sarcopenia and Muscle – Supplementary material. Supplementary references

Supplementary references

40. NS Pharma, Inc., Nippon Shinyaku Co. L. Study to Assess the Efficacy and Safety of Viltolarsen in Ambulant Boys With DMD (RACER53). Clinicaltrials.gov [www.clinicaltrials.gov]. 2021.

41. Pfizer. A Phase 3 Study to Evaluate the Safety and Efficacy of PF-06939926 for the Treatment of Duchenne Muscular Dystrophy. Clinicaltrials.gov [www.clinicaltrials.gov]. 2020.

42. ReveraGen BioPharma, Inc. EU, Group CINR, University N, Pittsburgh U of. A Study to Assess the Efficacy and Safety of Vamorolone in Boys With Duchenne Muscular Dystrophy (DMD) [Internet]. Clinicaltrials.gov [www.clinicaltrials.gov]. 2018 [cited 2021 Oct 5]. Available from: https://clinicaltrials.gov/ct2/show/NCT03439670?cond=Duchenne+Muscular+Dystrophy&age=0&draw=2&rank=1

43. Saoudi A, Zarrouki F, Sebrié C, Izabelle C, Goyenvalle A, Vaillend C. Emotional behavior and brain anatomy of the mdx52 mouse model of Duchenne muscular dystrophy. Dis Model Mech. 2021 Sep;14(9).

44. Vaillend C, Ungerer A. Behavioral characterization of mdx3cv mice deficient in C-terminal dystrophins. Neuromuscul Disord. 1999 Jul;9(5):296–304.

45. Lidov HG, Kunkel LM. Dp140: alternatively spliced isoforms in brain and kidney. Genomics. 1997 Oct;45(1):132–9.

46. Lidov HG, Selig S, Kunkel LM. Dp140: a novel 140 kDa CNS transcript from the dystrophin locus. Hum Mol Genet. 1995 Mar;4(3):329–35.

47. Chamova T, Guergueltcheva V, Raycheva M, Todorov T, Genova J, Bichev S, et al. Association between loss of dp140 and cognitive impairment in duchenne and becker dystrophies. Balkan J Med Genet. 2013 Jun;16(1):21–30.

48. Tennyson CN, Dally GY, Ray PN, Worton RG. Expression of the dystrophin isoform Dp71 in differentiating human fetal myogenic cultures. Hum Mol Genet [Internet]. 1996 [cited 2021 Apr 11];5(10):1559–66. Available from: https://pubmed.ncbi.nlm.nih.gov/8894689/

49. Kawaguchi T, Niba ETE, Rani AQM, Onishi Y, Koizumi M, Awano H, et al. Detection of Dystrophin Dp71 in Human Skeletal Muscle Using an Automated Capillary Western Assay System. Int J Mol Sci. 2018 May;19(6).

50. Beekman C, Janson AA, Baghat A, van Deutekom JC, Datson NA. Use of capillary Western immunoassay (Wes) for quantification of dystrophin levels in skeletal muscle of healthy controls and individuals with Becker and Duchenne muscular dystrophy. PLoS One [Internet]. 2018 Apr 1 [cited 2021 Apr 11];13(4). Available from: https://pubmed.ncbi.nlm.nih.gov/29641567/

51. Suárez-Sánchez R, Aguilar A, Wagstaff KM, Velez G, Azuara-Medina PM, Gomez P, et al. Nucleocytoplasmic shuttling of the Duchenne muscular dystrophy gene product dystrophin Dp71d is dependent on the importin α/β and CRM1 nuclear transporters and microtubule motor dynein. Biochim Biophys Acta. 2014 May;1843(5):985–1001.

52. Rapaport D, Greenberg DS, Tal M, Yaffe D, Nudel U. Dp71, the nonmuscle product of the Duchenne muscular dystrophy gene is associated with the cell membrane. FEBS Lett. 1993 Aug;328(1–2):197–202.

53. Leibovitz S, Meshorer A, Fridman Y, Wieneke S, Jockusch H, Yaffe D, et al. Exogenous Dp71 is a dominant negative competitor of dystrophin in skeletal muscle. Neuromuscul Disord. 2002 Nov;12(9):836–44.

54. Cox GA, Sunada Y, Campbell KP, Chamberlain JS. Dp71 can restore the dystrophin-associated glycoprotein complex in muscle but fails to prevent dystrophy. Nat Genet. 1994

Dec;8(4):333–9.

55. Greenberg DS, Sunada Y, Campbell KP, Yaffe D, Nudel U. Exogenous Dp71 restores the levels of dystrophin associated proteins but does not alleviate muscle damage in mdx mice. Nat Genet. 1994 Dec;8(4):340–4.

56. Howard PL, Dally GY, Ditta SD, Austin RC, Worton RG, Klamut HJ, et al. Dystrophin isoforms Dp71 and Dp427 have distinct roles in myogenic cells. Muscle and Nerve. 1999;22(1):16–27.

57. Farea M, Rani AQM, Maeta K, Nishio H, Matsuo M. Dystrophin Dp71ab is monoclonally expressed in human satellite cells and enhances proliferation of myoblast cells. Sci Rep [Internet]. 2020 Dec 1 [cited 2021 Apr 11];10(1). Available from: https://pubmed.ncbi.nlm.nih.gov/33051488/

58. Tennyson CN, Dally GY, Ray PN, Worton RG. Expression of the dystrophin isoform Dp71 in differentiating human fetal myogenic cultures. Hum Mol Genet. 1996 Oct;5(10):1559–66.

59. Howard PL, Dally GY, Ditta SD, Austin RC, Worton RG, Klamut HJ, et al. Dystrophin isoforms DP71 and DP427 have distinct roles in myogenic cells. Muscle Nerve. 1999 Jan;22(1):16–27.

60. Echigoya Y, Lee J, Rodrigues M, Nagata T, Tanihata J, Nozohourmehrabad A, et al. Mutation types and aging differently affect revertant fiber expansion in dystrophic mdx and mdx52 mice. PLoS One. 2013;8(7):e69194.

61. Desguerre I, Christov C, Mayer M, Zeller R, Becane H-M, Bastuji-Garin S, et al. Clinical heterogeneity of duchenne muscular dystrophy (DMD): definition of sub-phenotypes and predictive criteria by long-term follow-up. PLoS One. 2009;4(2):e4347.

62. Westendorp M, Hartman E, Houwen S, Smith J, Visscher C. The relationship between gross motor skills and academic achievement in children with learning disabilities. Res Dev Disabil [Internet]. 2011;32(6):2773–9. Available from: http://www.sciencedirect.com/science/article/pii/S0891422211002186

63. Battini R, Chieffo D, Bulgheroni S, Piccini G, Pecini C, Lucibello S, et al. Cognitive profile in Duchenne muscular dystrophy boys without intellectual disability: The role of executive functions. Neuromuscul Disord. 2018 Feb;28(2):122–8.

64. Hellebrekers DMJ, Doorenweerd N, Sweere DJJ, van Kuijk SMJ, Aartsma-Rus AM, Klinkenberg S, et al. Longitudinal follow-up of verbal span and processing speed in Duchenne muscular dystrophy. Eur J Paediatr Neurol EJPN Off J Eur Paediatr Neurol Soc. 2020 Mar;25:120–6.

65. Manto M, Bower JM, Conforto AB, Delgado-García JM, da Guarda SNF, Gerwig M, et al. Consensus paper: roles of the cerebellum in motor control--the diversity of ideas on cerebellar involvement in movement. Cerebellum [Internet]. 2012 Jun;11(2):457–87. Available from: https://pubmed.ncbi.nlm.nih.gov/22161499

66. Salman MS, Tsai P. The Role of the Pediatric Cerebellum in Motor Functions, Cognition, and Behavior: A Clinical Perspective. Neuroimaging Clin N Am. 2016 Aug;26(3):317–29.

67. von Haehling S, Morley JE, Coats AJS, Anker SD. Ethical guidelines for publishing in the journal of cachexia, sarcopenia and muscle: update 2017. Vol. 8, Journal of cachexia, sarcopenia and muscle. 2017. p. 1081–3.
